# Features of Peripapillary Hyperreflective Ovoid Mass-Like Structures in Nonarteritic Anterior Ischemic Optic Neuropathy Patients and Normal Controls

**DOI:** 10.1167/tvst.13.1.7

**Published:** 2024-01-12

**Authors:** Wenyu Wang, Juejun Liu, Di Xiao, Zuohuizi Yi, Changzheng Chen

**Affiliations:** 1 Department of Ophthalmology, Renmin Hospital of Wuhan University, Wuhan, China

**Keywords:** peripapillary hyperreflective ovoid mass-like structure, nonarteritic anterior ischemic optic neuropathy, Bruch's membrane opening, optical coherence tomography angiography, normal participants

## Abstract

**Purpose:**

To determine the characteristics of peripapillary hyperreflective ovoid mass-like structures (PHOMS) in patients with nonarteritic anterior ischemic optic neuropathy (NAION) and in normal adults.

**Methods:**

A total of 406 included eyes were divided into four groups: acute NAION group, chronic NAION group, unaffected group, and normal eyes group. PHOMS were detected on optical coherence tomography slices from optical coherence tomography angiography scans centered on the optic nerve head (ONH). The differences in age, sex, and ONH parameters were investigated between eyes with PHOMS and eyes without PHOMS among groups.

**Results:**

The prevalence of PHOMS in acute eyes (43.48%) and fellow eyes (28.20%) was significantly higher than that in normal eyes (11.76%) (acute vs. normal, *P* < 0.001; fellow vs. normal, *P* = 0.014). In the acute group, the PHOMS score of size was negatively correlated with age in acute eyes (*r* = −0.486, *P* = 0.03). The size of PHOMS was negatively correlated with age and cup/disc ratio and positively correlated with retinal nerve fiber layer thickness in the nasal and inferior sectors in the normal groups. No differences in age, sex, ONH parameters, or visual field defects were found between eyes with PHOMS and eyes without PHOMS.

**Conclusions:**

The prevalence of PHOMS increased significantly in acute nonoptic disc drusen (NODD)–NAION eyes and fellow eyes. PHOMS could also be found among normal adults. PHOMS may be a nonspecific sign secondary to ONH edema and axoplasmic stasis.

**Translational Relevance:**

The high prevalence of PHOMS in acute NODD-NAION eyes may indicate axoplasmic stasis secondary to tissue edema.

## Introduction

Due to the clinical application of enhanced depth imaging optical coherence tomography (OCT), peripapillary hyperreflective ovoid mass-like structures (PHOMS) have gradually attracted attention. In a previous study, PHOMS have been confused with optic disc drusen (ODD).^1^^–^^3^ The Optic Disc Drusen Studies Consortium defined PHOMS as a separate entity from ODD.^4^ PHOMS are located next to the optic disc and above Bruch's membrane opening (BMO). In addition, their reflection is similar to that of the retinal nerve fiber layer (RNFL). Histopathologic studies of the optic nerve head (ONH) in papilledema have shown distended and vacuolated optic nerve axons anterior to the lamina cribrosa, particularly in the peripapillary part of the nerve.^5^^,^^6^ The location and morphology of the structures corresponded exactly to PHOMS in disc edema, which were detected on optical coherence tomography (OCT).^5^^,^^6^ PHOMS have been observed in many conditions, including ODD,^7^ tilted disc syndrome,^8^^,^^9^ pseudopapilledema,^7^ myopia,^10^ central and branch retinal vascular occlusions,^11^ nonarteritic anterior ischemic optic neuropathy (NAION),^12^^,^^13^ multiple sclerosis (MS),^14^ papilledema,^15^ and neuromyelitis optic spectrum disease.^16^ Currently, PHOMS have been categorized by etiology: (1) disc edema-associated PHOMS, (2) ODD-associated PHOMS, and (3) anomalous disc-associated PHOMS.^17^^,^^18^ PHOMS appear as a cylindrical extent or toroidal extent on consecutive OCT slices.^17^^,^^19^ However, the histologic origin and mechanism of PHOMS are still unclear. As a biomarker of OCT imaging, further studies are required to determine the value of PHOMS in the diagnosis and treatment of diseases.

NAION is the most common cause of sudden optic nerve–related vision loss and typically occurs in individuals over 55 years of age.^20^ According to previous research, 7.5% to 23% of patients with NAION are younger than 50 years.^21^^–^^23^ At the onset of visual loss, optic disc edema is always present. Histopathologic study also found that the optic disc in NAION was characterized by edema, loss of cellularity, distention of nerve fiber bundles in the retrolaminar area, and distention of the collagen lamellae of the lamina cribrosa plate.^24^ There are numerous systemic risk factors associated with NAION, such as high blood pressure, hyperlipidemia, atherosclerosis, diabetes mellitus, episodic low blood pressure, and obstructive sleep apnea.^18^^,^^22^^–^^25^ However, local risk factors such as a small and crowded “disc at risk” also play a role in NAION, especially in relatively young patients.^23^^,^^25^ ODD is a calcified deposit localized in the axons of the prelaminar ONH.^26^^,^^27^ Many studies have found a higher incidence of ODD in young patients with NAION , and ODD has been considered an isolated risk factor for NAION.^13^^,^^23^^,^^28^ A high incidence of PHOMS has also been observed among patients with ODD-NAION .^13^ However, the clinical significance of PHOMS in patients with NAION remains uncertain.

The purpose of the study was to determine the prevalence of PHOMS in non-ODD NAION (NODD-NAION) patients and the influence of PHOMS on visual function and outcomes. The study also aims to examine the prevalence of PHOMS in normal adults without any ocular disease and investigate the association between the ONH structure and PHOMS.

## Materials and Methods

### Participants

 This retrospective study reviewed 470 eyes from 60 patients with NAION and 179 normal participants. A total of 406 eyes from 52 patients with NAION and 153 normal participants were ultimately included. Examinations were performed in 2018–2019 and 2021–2022 at Renmin Hospital of Wuhan University. The study was approved by the Institutional Review Board of the Renmin Hospital of Wuhan University (WDRY2023-K025) and conducted in accordance with the tenets of the Declaration of Helsinki, and informed consent was obtained from all participants. The included eyes were divided into four groups: (1) acute NAION group (*n* = 46 eyes), (2) chronic NAION group (*n* = 15 eyes), (3) unaffected NAION group (fellow normal eyes from patients with NAION) (*n* = 39 eyes), and (4) normal group (*n* = 306 eyes).

A diagnosis of NAION was made based on patients’ symptoms, clinical examinations, and fundus imaging, including visual acuity, visual field (VF), intraocular pressure, fundus fluorescein angiography , visual evoked potential , and color fundus photography. Magnetic resonance imaging was also performed to rule out papilledema induced by neurologic diseases. The erythrocyte sedimentation rate and C-reactive protein tests were performed to exclude arteritic ischemic optic neuropathy in all participants. Acute NAION was defined as a symptom duration of less than 2 weeks. The chronic NAION group included patients who were diagnosed with NAION for more than 8 weeks. The normal participants received optometry examination, best-corrected visual acuity (BCVA), ultrawide-field fundus photography, macular OCT, and ONH optical coherence tomography angiography (OCTA). ODDs on OCT scans are recognized according to the definition of the optic disc drusen studies (ODDS) consortium as hyporeflective structures with a full or partial hyperreflective margin on OCT B-scans.

### Inclusion Criteria

 Patients with NAION: (1) monocular acute visual loss with optic disc edema; (2) typical horizontal VF defect; (3) no signs of giant cell arteritis, including elevated erythrocyte sedimentation rate and C-reactive protein levels; (4) no signs of neurologic diseases; and (5) examination time less than 2 weeks or more than 8 weeks from the onset of visual symptoms.

Normal patients: (1) BCVA ≥ 20/20, (2) absence of ophthalmic disease history, and (3) normal fundus examinations.

### Exclusion Criteria

Patients with NAION: (1) participants with other ocular diseases (glaucoma, uveitis, pathologic myopia, central serous chorioretinopathy, optic neuritis, etc.), (2) OCTA images with poor quality (scan quality <5 or obvious motion artifact), (3) participants with ODD in either affected eye or the fellow eye, and (4) participants with refractive error >3.0 diopters (D) or <−6.0 D.

Normal patients: (1) participants with ophthalmic surgery and trauma history, (2) patients with severe systematic disease, (3) OCTA images with poor quality (scan quality <5 or obvious motion artifact), (4) participants with ODD, and (5) participants with refractive error >3.0 D or <−6.0 D.

### Image and Data Acquisition

OCTA images covering an area of 4.5 × 4.5 mm centered on the optic disc were acquired at three sessions using a commercial spectral domain OCT system with a scan rate of 70,000 A-scans per second (Avanti RTVue-XR; Optovue, Fremont, CA, USA) in the NAION group. The peripapillary RNFL thickness was evaluated at the peripapillary region and in four sectors using ONH analysis software (Angio DiscVue, Optovue, Fremont, CA, USA). The vessel density (VD) of the radial peripapillary capillary (RPC) was automatically calculated by the built-in software of the device. In the normal group, OCTA images were acquired using a commercial OCT instrument (VG100 ; SVision Imaging, Ltd., Luoyang, China) equipped with a 1050-nm-wavelength laser. OCTA was performed using a raster scan protocol of 512 (horizontal) × 512 (vertical) that covered an area of 6 × 6 mm centered on the ONH. RNFL thickness, VD of RPC, and cup/disc ratio (CDR) were evaluated at the peripapillary region by built-in software, version 1.36.4.

### Image Review and Analysis

**Figure 1.**
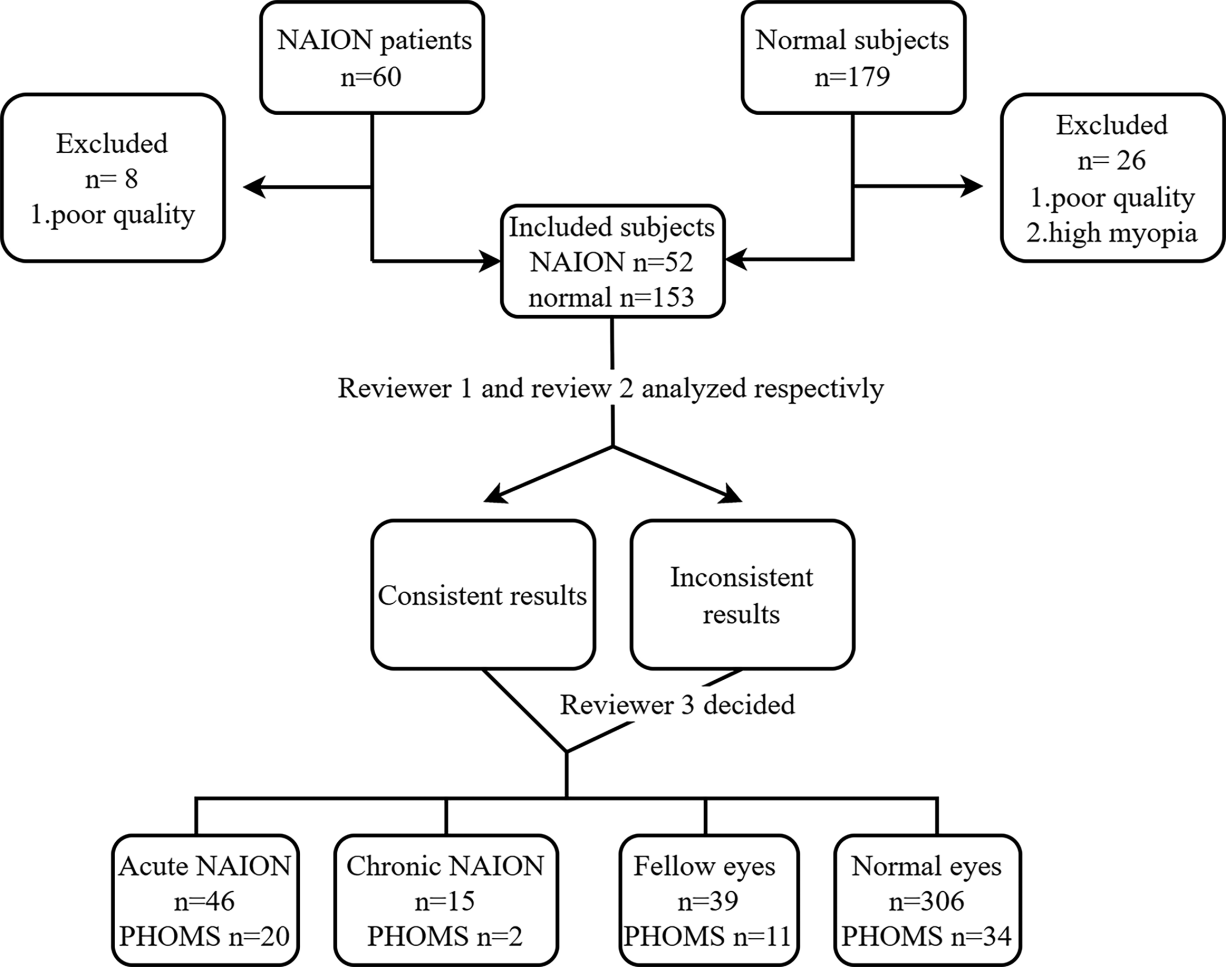
The process of patient inclusion and data analysis.

Two trained graders ( WW and JL) assessed all the B-scans from OCTA imaging of all the included participants. Consistent with previous studies, PHOMS were defined as homogeneous hyperreflective mass-like structures located in the peripapillary area immediately on top of Bruch's membrane.^9^^,^^17^ In the current study, PHOMS were considered present when they could be observed on five or more consecutive B-scans. Inconsistent results between the two graders were further analyzed by a senior retina specialist (CC). Figure 1 shows the process of the study.

**Figure 2.**
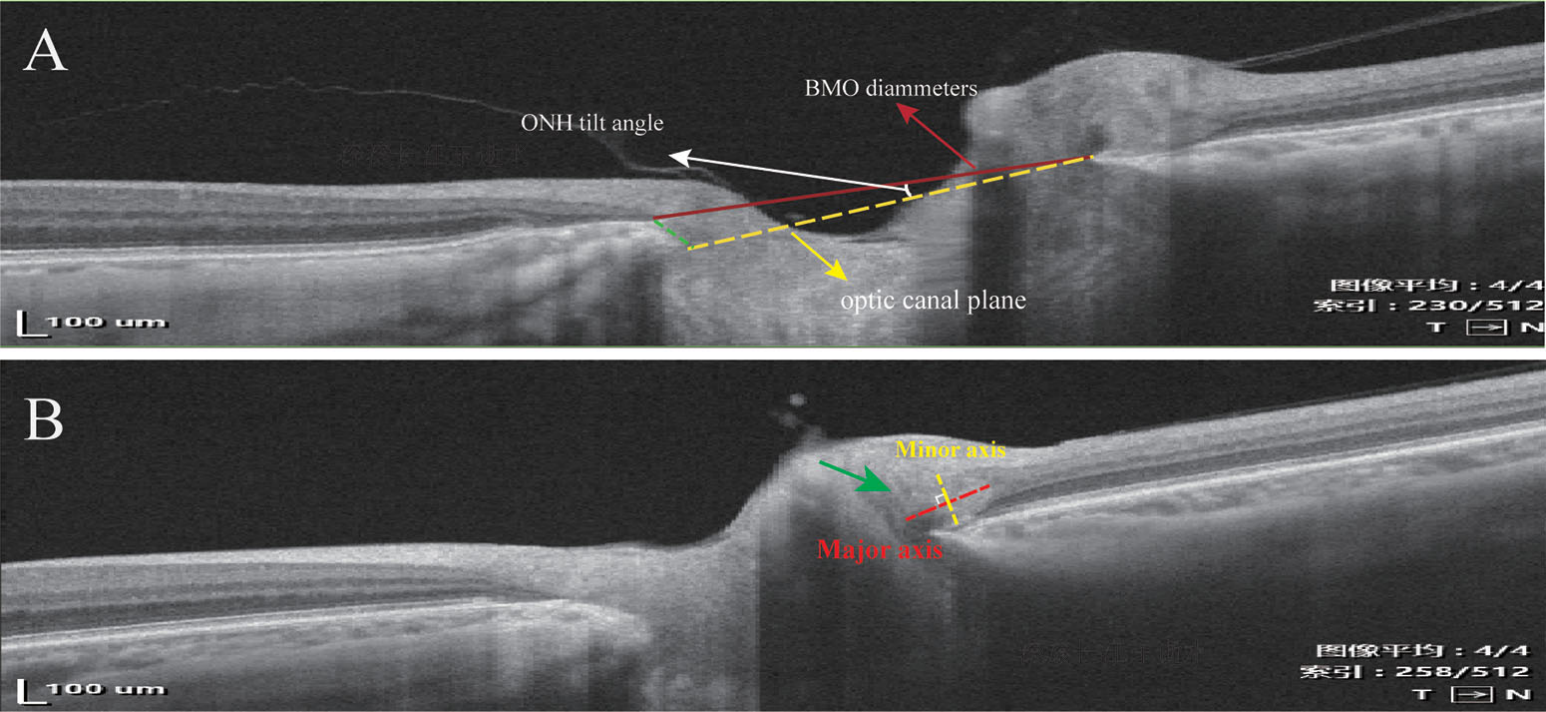
The example of ONH parameters and PHOMS size analysis. B-scans are all adjusted for scale. (**A**) *Red line* is the BMO plane. *Yellow dotted line* is optic canal plane. The angle between the BMO plane and optic canal plane is the ONH tilt angle. (**B**) PHOMS (*green arrow*) is subelliptical on B -scans. Major axis (*red dotted line*) is the longest axis of PHOMS and minor axis (*yellow dotted line*) is vertical to major axis.

BMO diameter and ONH tilt angle were measured by WW on scaled B-scan slices with the largest BMO diameter on ImageJ software (National Institutes of Health, Bethesda, MD, USA). BMO diameter is the distance between the nasal and temporal BMO, and ONH tilt was defined as the angle between the BMO plane and the optic canal plane (Fig. 2).^29^ PHOMS were quantified by major axis length and minor axis length. The largest major and minor axis lengths on B-scans of each PHOMS were considered a score of size and used for statistical analysis (Fig. 2).

### Statistical Analysis

We used SPSS Statistics (version 26.0; IBM Corp., Armonk, NY, USA). Continuous variables with a normal distribution are presented as the mean ± standard deviation. Continuous variables that did not follow a normal distribution are presented as the median (interquartile range). After using the Shapiro–Wilk test to test the normality of the data and Levene's test for the homogeneity test of variance, independent samples *t*-tests or independent samples Mann–Whitney *U* tests were used to compare age and ONH parameters between eyes with PHOMS and eyes without PHOMS in the acute NAION and the unaffected eye groups. Considering that bilateral eyes of the normal group were included, generalized estimating equations were used to eliminate the effect of repeatability and relevance in data in the normal group. Chi-square tests or Fisher’s precision probability tests were used to compare sex differences between eyes with PHOMS and eyes without PHOMS. Based on the data distribution type, the Pearson correlation analysis or the Spearman correlation analysis was used to verify the correlation between the PHOMS size and ONH parameters. Differences were considered statistically significant at *P* < 0.05.

## Results

In the current study, 48.08% (*n* = 25) of patients with NAION had PHOMS, with 8 (32%) patients bilateral and 17 (68%) patients unilateral. Among 17 patients with unilateral PHOMS, PHOMS were present in 12 NAION eyes and 5 normal fellow eyes. In total, 18.9% (*n* = 29) of normal participants had PHOMS, with 5 (17.24%) participants bilateral and 24 (82.76%) patients unilateral. To investigate the characteristics of the PHOMS eyes from 4 groups, we chose the right eyes of the normal participants and compared them with the acute NAION group, the chronic NAION eyes, and the fellow normal eyes from the patients with NAION. The specific results are shown in Table 1. The prevalence of PHOMS in acute eyes and fellow eyes was significantly higher than that in the normal group. In addition, the major axis and the minor axis of PHOMS were statistically longer in acute NAION eyes, which indicated that PHOMS in acute NAION eyes were larger than in the fellow group or normal group. We also investigated the location of PHOMS in four groups. More PHOMS were found in the nasal and inferior peripapillary regions, and no difference in location was found between the groups.

**Table 1.** Comparison among Eyes with PHOMS in Different Groups

Characteristic	Normal^a^	Unaffected	Acute	Chronic	*P*	*P*1	*P*2	*P*3
*N*	18	11	20	2				
Incidence, %	11.76	28.20	43.48	14.29	**<** **0.001**	0.178	**0.001**	**0.014**
Age, y	51.78 ± 14.64	56.00 ± 10.11	53.00 ± 7.11	57.50 ± 7.78	0.728			
Major axis, µm	433.40 ± 266.26	407.05 ± 208.1	633.23 (396.57)	457.04 ± 383.86	**0.017**	0.084	**0.032**	1
Minor axis, µm	181.50 ± 53.78	203.99 ± 78.09	298.52 ± 116.73	230.20 ± 98.84	**0.002**	**0.035**	**0.001**	0.913
Location, %								
Nasal	55.6	54.5	65	0	0.44			
Temporal	22.2	27.3	40	100	0.166			
Superior	44.4	45.5	40	50	0.932			
Inferior	61.1	54.5	60	50	0.798			

Major axis, the major axis length of PHOMS on B-scan with the largest cross-sectional area; Minor axis, the axis length vertical to the major axis; *P*1, compared between acute NAION eyes and the unaffected eyes; *P*2, compared between acute NAION eyes and normal eyes; *P*3, compared between unaffected eyes and normal eyes. The bold values represent the statistical significance.

a Only includes the right eye from normal patients for comparison among groups.

**Figure 3.**
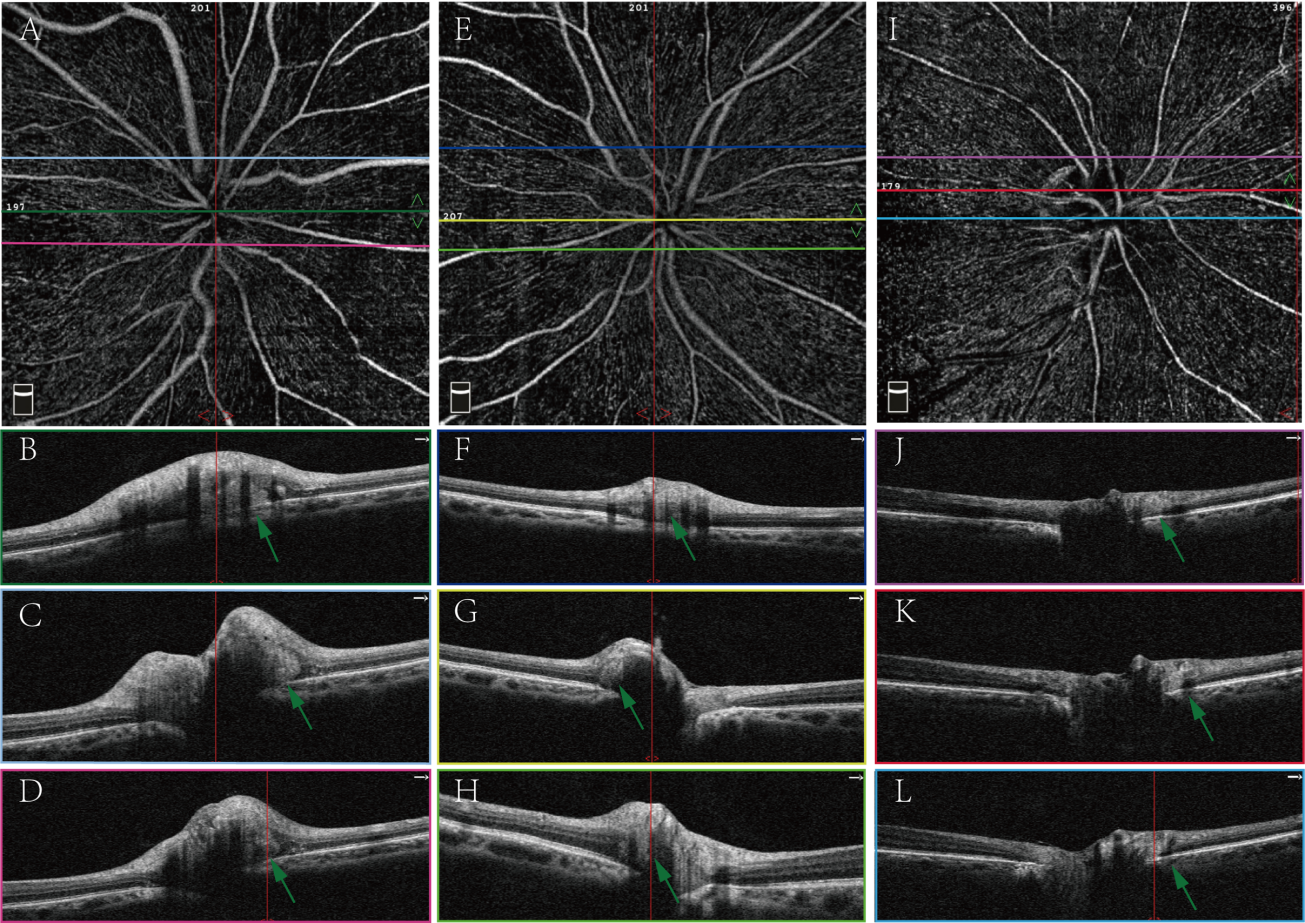
The example of PHOMS in patients with NAION. (**A–D** ) OCTA images from an acute NAION eye (patient M). PHOMS could be found in multiple B-scans in superior and nasal peripapillary region (*green arrows* in **B**, **C**, and **D**). **B**, **C**, and **D** are corresponding to the lines with the same colors in **A**. (**E–H**) OCTA images from the normal fellow eye of patient M. PHOMS are shown in *green arrows* in **F**, **G**, and **H**. **F**, **G**, and **H** are corresponding to the lines with the same colors in **E**. (**I–L** ) OCTA images from a chronic NAION eye (patient G, disease duration was 10 weeks). PHOMS could be found in nasal peripapillary region on B-scans (*green arrows* in **J–L**). **J**, **K**, and **L** are corresponding to the lines with the same colors in **I**.

**Figure 4.**
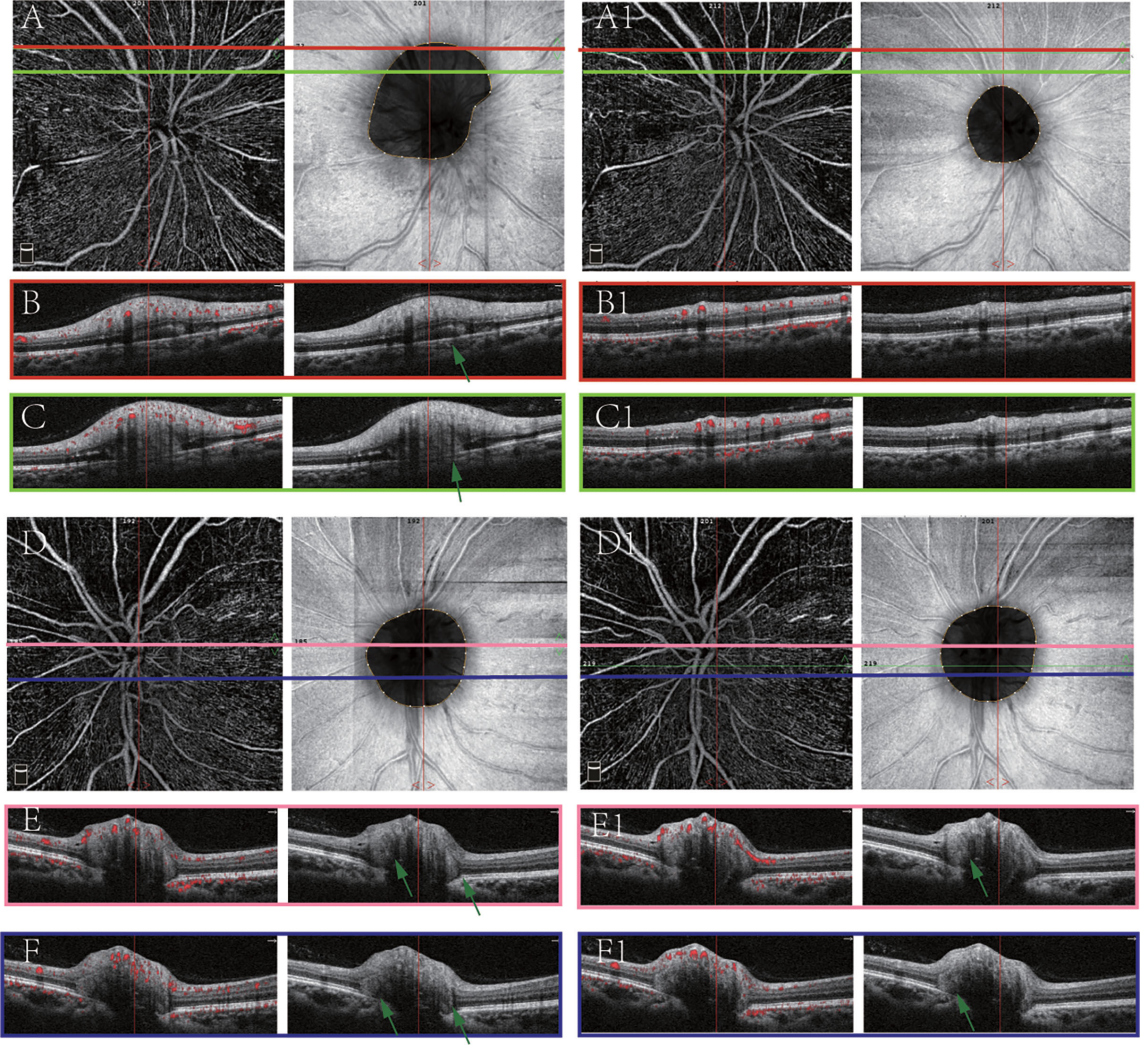
PHOMS in the acute NAION eye resolved in the follow-up period. **A–C** and **D–F** are images from the NAION eye and fellow normal eye, respectively, at baseline (5 days from onset). PHOMS could be found in the superior peripapillary region in the NAION eye (*green arrows* in **B** and **C**). PHOMS could be found in nasal and temporal regions in the fellow eye (*green arrows* in **E** and **F**). **A1–C1** and **D1–F1** are images acquired after 8 weeks. The atrophy of ONH could be found in the affected eye. PHOMS in the corresponding locations resolved (**B1** and **C1**). In the fellow eye, the location and morphology of PHOMS remained stable after 8 weeks (**E1** and **F1**).

**Figure 5.**
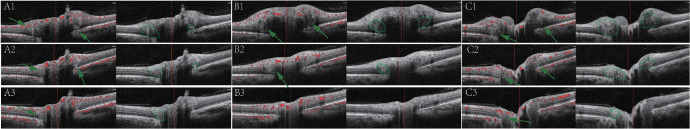
PHOMS persisted in an NAION eye for 12 weeks. **A1**, **B1**, and **C1** are OCT slices from one patient in different locations at baseline (acquired within 7 days after disease onset). PHOMS could be found in nasal and temporal peripapillary regions. **A2**, **B2**, and **C2** were acquired 31 days from the first visit. **A3**, **B3**, and **C3** were acquired 12 weeks from baseline. During the follow-up, PHOMS locating in the nasal region resolved. However, PHOMS in the temporal side were persistent. PHOMS are marked with *green arrows* and *dotted green circles*.

We further compared ONH parameters between eyes with PHOMS and eyes without PHOMS. The results of acute NAION eyes and fellow eyes are shown in the Supplementary Table S1. There was no difference in age, sex, RNFL thickness, VD of RPC, BMO, ONH tilt angle, or VF between eyes with PHOMS and eyes without PHOMS in the two groups. Examples of NAION eyes with PHOMS are shown in Figure 3. Nine patients had follow-up data, and the median follow-up period was 8 weeks. Among the follow-up eyes, five acute NAION eyes had PHOMS. PHOMS in the acute stage disappeared after 4 to 8 weeks of onset (Fig. 4), except that the PHOMS of one patient still persisted 21 weeks after onset (Fig. 5). In addition, PHOMS in two normal fellow eyes remained stable during the follow-up period (Fig. 4). In the telephone interview (8 months from disease onset), NAION did not occur in all unaffected eyes of 29 patients, whether with PHOMS or without PHOMS. In NAION eyes with PHOMS, we further investigated the relationship between the PHOMS size and other parameters. The results are shown in Table 2. We only found that the PHOMS score of size was negatively correlated with age in acute eyes. No correlation was found in fellow eyes (specific results are shown in Supplementary Table S2).

**Table 2.** Correlation Analysis Between the Size of PHOMS and Potential Factors in the Acute NAION Group

Characteristic	*r*	*P* ^a^
Age	−0.486	**0.03**
BMO	−0.138	0.36
ONH tilt angle	0.275	0.065
VF (MD)	−0.184	0.451
RNFL thickness		
Superior	−0.094	0.584
Nasal	−0.129	0.453
Inferior	−0.031	0.856
Temporal	−0.117	0.495

MD, mean deviation. The bold values represent the statistical significance.

a Statistical analysis is calculated with Pearson correlation analysis or Spearman correlation analysis.

**Figure 6.**
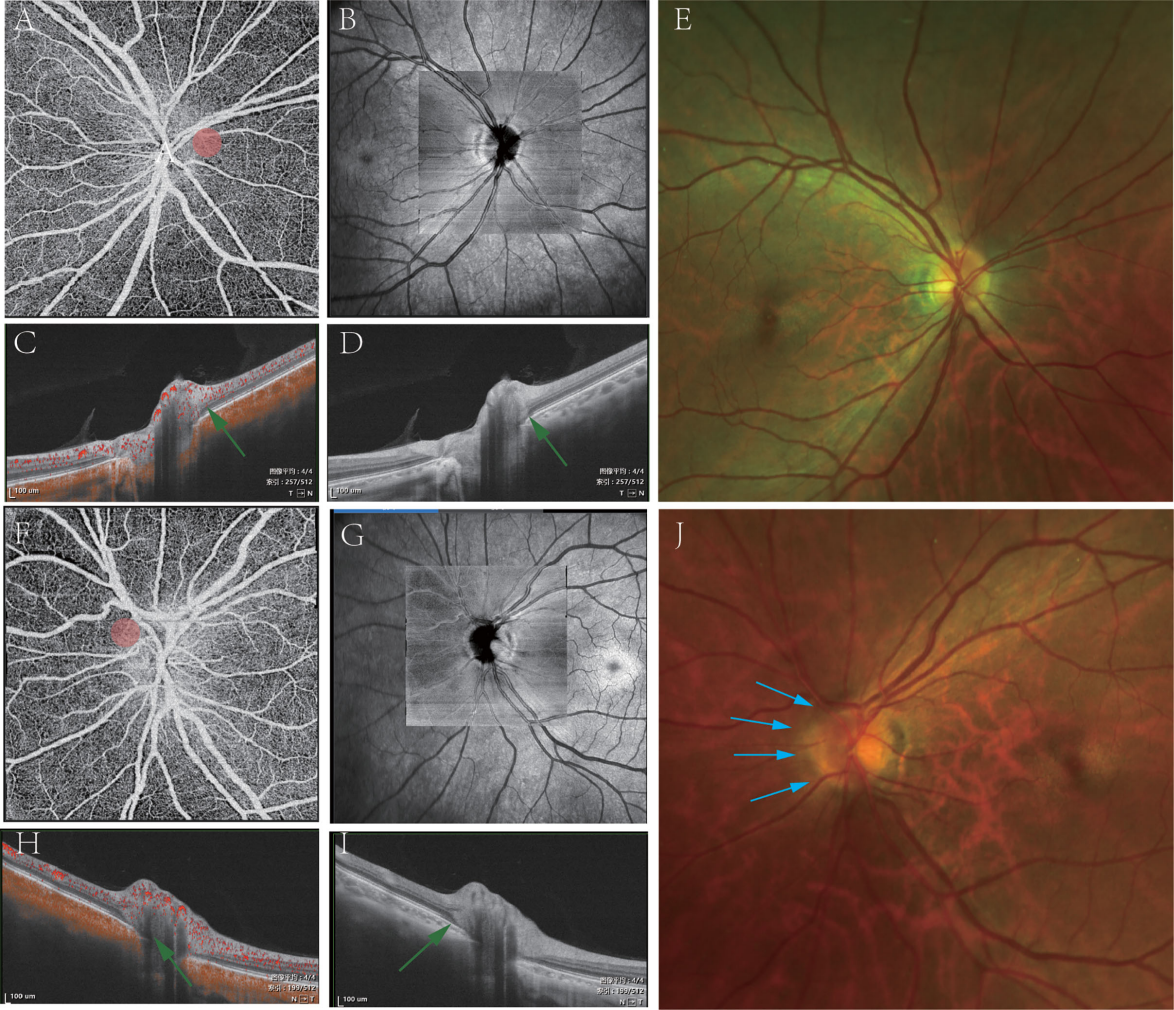
The example of PHOMS in normal patients. The participant is a 52-year-old woman without any retinal disease. PHOMS were present in nasal peripapillary regions in both eyes (*green arrows* in **D** and **I**). *Pink circles* in **A** and **F** represent the location of PHOMS. Small blood flow signals can be found in **C** and **H**. The ONH boundary is clear on the right on infrared imaging (**B**) and fundus photography (**E**). The nasal boundary of ONH is blurred in the left eye on infrared imaging (**G**) especially in fundus photography (*blue arrows* in **J**).

In normal participant groups, there was also no difference in demographic characteristics or ONH parameters, including age, sex, RNFL thickness, VD of RPC, BMO, ONH tilt angle, or CDR, between eyes with PHOMS and eyes without PHOMS (specific results are shown in Supplementary Table S3). In the correlation analysis, we found that the PHOMS score of size was negatively correlated with age and CDR. The PHOMS size was also positively correlated with RNFL thickness in the nasal and inferior regions. The results are shown in Table 3. In partial correlation analysis, only the average RNFL thickness of the nasal and inferior sectors was positively correlated with the PHOMS size. The results of multiple linear regression analysis are shown in Table 4. Examples of normal eyes with PHOMS are shown in Figure 6.

**Table 3.** Correlation Analysis Between the Size of PHOMS and Potential Factors in Normal Participants

Characteristic	*r*	*P* ^a^
Age	−0.461	**0.007**
BMO	0.045	0.805
ONH tilt angle	0.012	0.946
Retina thickness		
Superior	0.298	0.092
Nasal	0.59	**<** **0.001**
Inferior	0.494	**0.003**
Temporal	0.116	0.521
CDR	−0.456	**0.008**

The bold values represent the statistical significance.

a Statistical analysis is calculated with Pearson correlation analysis or Spearman correlation analysis.

**Table 4.** Multiple Linear Regression Analysis in Normal Participants

Variable	β	*P*
Age	0.028	0.893
RNFL thickness	0.404	**0.026**
CDR	−0.338	0.12

*F* = 6.348, *P* = 0.002, *R*^2^ = 0.396. RNFL thickness = nasal RNFL thickness + temporal RNFL thickness. The bold values represent the statistical significance.

## Discussion

In the current study, we reported the prevalence of PHOMS in normal adults and patients with NAION without ODD. We also investigated the relationship between the ONH parameters and the size of PHOMS and observed the changes in PHOMS in patients with acute NAION with the course of disease. Based on our review of the PubMed and Web of Science, we believe that the study is the first to assess the prevalence of PHOMS in normal adults without ODD and patients with NAION without ODD and to analyze the characteristics of ONH morphology with PHOMS.

In the NAION group, we found an increased prevalence (43.48%) of PHOMS in acute NODD-NAION eyes. Johannesen et al.^12^ found that patients with ODD-NAION had significantly more PHOMS than those without NODD-NAION. Another study reported that 28% of eyes with NODD-NAION had PHOMS, whereas 54% with ODD-NAION had PHOMS.^13^ Compared with the normal group (11.76%) and the unaffected group (28.20%), the prevalence of PHOMS increased significantly in the acute NAION group (43.48%). In addition, PHOMS in the acute group were larger than those in the other two groups. A histopathologic study of ischemic optic neuropathy showed optic disc swelling with peripapillary crowding of the retina resembling PHOMS.^24^ Our result is consistent with the current opinions that PHOMS are associated with ONH edema secondary to various causes, including NAION,^30^ even though there was no ODD present. In our study, most PHOMS tended to disappear with the resolution of ONH edema. However, PHOMS persisted in one patient for more than 5 months. PHOMS were found in two chronic NAION. Petzold et al.^31^ found that most PHOMS in patients with MS remained stable, although an increase in size was observed in some patients. One possible explanation for the findings from the acute NAION group is that axoplasmic stasis secondary to tissue edema caused the increased prevalence and larger size of PHOMS. As the axoplasmic stasis resolved, most PHOMS disappeared. However, the relationship between the presence of PHOMS and functional abnormalities cannot be determined in the current study. In the statistical analysis, age and local anatomic parameters of the ONH had no influence on the presence of PHOMS. PHOMS had no effect on VF defects in acute NAION eyes.

Another interesting finding is that the prevalence of PHOMS is higher in unaffected fellow eyes of patients with acute NAION than in normal eyes. Most PHOMS persisted and remained stable in the unaffected eyes, which was not different from the NAION eyes. The relatively high prevalence of PHOMS may be related to crowded discs in unaffected eyes, which is considered a local risk factor for NAION.^23^ Although in the unaffected group, eyes with PHOMS showed shorter BMO diameters and larger ONH tilt angles than eyes without PHOMS, the difference was not statistically significant. During the follow-up period, the fellow eyes remained unaffected in eyes with and without PHOMS. The available evidence does not support that the presence of PHOMS is a risk factor for NAION. More prospective studies are needed to determine the clinical significance of PHOMS in the morbidity of NAION.

According to recent studies, PHOMS have been classified as disc edema-associated PHOMS, ODD-associated PHOMS, or anomalous disc-associated PHOMS.^17^ Our study illustrated that PHOMS can also occur in normal eyes without abnormal ONH morphology. A total of 18.9% of normal patients had PHOMS, and 17.24% of them had bilateral PHOMS. A previous study of patients with MS with PHOMS found that there were no PHOMS in healthy controls.^31^ A population-based study found that 8.9% of normal children aged 11 to 12 years had PHOMS.^9^ PHOMS were also considered a reason for pseudopapilledema.^7^^,^^32^^,^^33^ By reviewing fundus photography, we found that most discs showed normal morphology and a clear margin in the normal group. One possible reason for the normal disc morphology is that most PHOMS in the normal group were small and did not contribute to the obvious change in the appearance of the ONH. Among normal individuals, we found a positive correlation between the maximum cross-sectional area and nasal and inferior RNFL thickness, which corresponds to the location of PHOMS. The maximum cross-sectional area of PHOMS was also negatively correlated with age and CDR. The correlation analysis supported that local factors of ONH played a role in the formation of PHOMS.

Pathologic evidence suggests that PHOMS represent lateral bulges of nerve fibers.^27^^,^^34^ Recently, some studies with OCTA revealed blood flow signals in these hyperreflective lesions, which may indicate that there are vascular structures within PHOMS.^35^^,^^36^ Researchers have also found a case in which vascularized PHOMS were connected to peripapillary subretinal neovascularization. In our study, blood flow signals in PHOMS were similar to those in RNFL layers. We speculate that there are capillary vessel structures in PHOMS.^24^^,^^27^ The results in our study do not support the opinion from Borrelli et al.^35^ that the presence of PHOMS might bring deeper vessels deputed at the irroration of the optic nerve into the retina.^24^ The clinical significance of blood flow in PHOMS needs to be further investigated.

There are some limitations in the current study. First, the included OCTA scans of patients with NAION were acquired on spectral domain OCT (SD-OCT), and OCTA scans of normal participants were acquired on swept source OCT in the retrospective study. Considering that PHOMS are superficially located above the BMO, SD-OCT may not contribute to the decreased detectability of PHOMS. Second, in quantitative analysis, we used the maximum sectional area to estimate the volume of PHOMS. The quantitative methods used in the study may be inaccurate enough in analyzing three-dimensional structures. Although Jorgensen et al.^37^ developed a method to calculate the volume of PHOMS, it could only be applicable in radial OCT B-scans on ONH. In future studies, machine learning can be trained to detect PHOMS on every single B-scan and determine the true volume of PHOMS. Third, the relationship between PHOMS and axial lengths was not investigated due to incomplete data. In the study, we excluded participants with high myopia or hyperpresbyopia. Lyu et al.^10^ found an association between myopia and PHOMS in children. Many studies have illustrated that the morphology of the ONH changes with increasing axial length.^38^^–^^40^ Axial length is an important factor in ONH deformation, and studies in the future need to take it into consideration. Last but not least, although PHOMS could be found in normal individuals and unaffected fellow eyes of NAION, follow-up data are insufficient in our study. Further prospective studies based on populations could illuminate the changes in PHOMS in the long term and their clinical significance.

In general, we found an increased prevalence of PHOMS with a larger volume in acute NODD-NAION eyes. Most PHOMS were eliminated along with the resolution of ONH edema. The prevalence of PHOMS in NAION had no effect on functional and structural outcomes. The current study is consistent with the previous perspective that PHOMS are a common but nonspecific OCT marker secondary to axoplasmic stasis in the edematous ONH.^17^ PHOMS also occurred in normal participants and unaffected normal eyes from patients with NAION, most of whom had a normal appearance of ONH without ODD. The investigation of PHOMS may provide new insight into the morphologic and structural characteristics of ONHs.

## Supplementary Material

Supplement 1
