# Supplementary material for: Features of Peripapillary Hyperreflective Ovoid Mass-Like Structures in Nonarteritic Anterior Ischemic Optic Neuropathy Patients and Normal Controls
Source: Transl Vis Sci Technol. 2024 Jan 12;13(1):7. doi: 10.1167/tvst.13.1.7 (PMC10790673; doi:10.1167/tvst.13.1.7)
Supplement: Supplement 1 [file tvst-13-1-7_s001.pdf]

Table S1 Comparison between eyes with PHOMS and without PHOMS in unaffected group

	with PHOMS	without PHOMS	P[△]
n (%)	11 (28.2)	28 (71.8)	
gender (female, %)	4 (36.36)	7 (25)	0.694[*]
Age	55.45±10.27	54.96±6.01	0.884
BMO diameter(μm)	1845.14±348.88	1978.44±209.43	0.15
ONH tilt (°)	3.64±3.58	2.83±1.82	0.488
VD of RPC (%)	62.75±0.21	58.35±2.89	0.059
RNFL (μm)			
Superior	143.11±36.74	138.04±15.99	0.586
Temporal	86.33±13.24	82.52±13.30	0.645
Inferior	157.00 (40)	140.00 (25)	0.301[*]
Nasal	94.00±4.24	82.57±11.56	0.471
VF (MD, db)	-4.09 (4.85)	-4.09 (3.21)	0.964[*]
missing	6	14	

BMO, bruch’s membrane opening; ONH, optic nerve head; VD, vessel density; RPC, RPC, peripapillary radical capillary; RNFL, retinal nerve fiber layer; VF, visual field; MD, mean deviation

[△] Statistical significance was calculated with independent-samples T test except for special labe

[*]Statistical significance was calculated with Mann-Whitney U test

[*]Statistical significance was calculated with Fisher's exact test

Table S2 Comparison between eyes with PHOMS and without PHOMS in acute NAION group

	with PHOMS	without PHOMS	P[Δ]
n (%)	20 (43.48)	26 (56.52)	
gender			
(female, %)	9 (45)	7 (26.92)	0.229[※]
Age	53.00±7.11	56.35±8.13	0.152
BMO (μm)	2197.03±274.76	2154.90±292.20	0.621
ONH tilt (°)	1.62±1.47	2.15±2.78	0.445
VD of RPC (%)	52.80 (8)	54.30 (10)	0.366*
RNFL (μm)			
Superior	209.82±69.44	199.37±54.89	0.618
Temporal	140.53±54.68	156.32±51.58	0.379
Inferior	196.35±61.30	214.11±56.504	0.372
Nasal	148.65±67.01	141.05±40.71	0.68
VF (MD, db)	‘-18.33±7.80	‘-18.40±5.65	0.981
missing	13	10	

BMO, bruch’s membrane opening; ONH, optic nerve head; VD, vessel density; RPC, RPC, peripapillary radical capillary; RNFL, retinal nerve fiber layer; VF, visual field; MD, mean deviation

[Δ] Statistical significance was calculated with independent-samples T test except for special label

[*]Statistical significance was calculated with *Mann-Whitney U test*
[※]Statistical significance was calculated with Fisher's exact test

Table S3 Comparison between eyes with PHOMS and without PHOMS in normal group

	with PHOMS	without PHOMS	P^
n (%)	34 （11.11）	272 （88.88）	
gender (female, %)	50.76±13.20	51.62±11.83	0.875
Age	51.00（19）	23.00（18）	0.734
BMO (μm)	1859.243±235.49	1878.29±211.87	0.677
ONH tilt (°)	3.78（4.81）	3.63 (4.11)	0.929
VD of RPC (%)	61.84 (7.39)	61.41 (7.91)	0.733
RNFL (μm)			
Superior	135.50 (33)	136.00 (32.75)	0.468
Temporal	81.00 (19.50)	81.00 (19.00)	417
Inferior	144.50 (33.50)	141.00 (27.00)	0.218
Nasal	81.00 (24.00)	83.00 (21.00)	0.409
CDR	0.48 (0.53)	0.43 (0.47)	0.29

BMO, bruch's membrane opening; ONH, optic nerve head; VD, vessel density; RPC, RPC, peripapillary radical capillary; RNFL, retinal nerve fiber layer; VF, visual field; MD, mean deviation; CDR, cup-disc ratio

^ Statistical significance is calculated with Generalized Estimation Equations

Correlation analysis between the size of PHOMS and potential factors in unaffected fellow group

	r	P▲
Age	-0.214	0.527
BMO	-0.1	0.77
ONH tilt angle	-0.227	0.502
VF (MD)	0.1	0.873
retina thickness		
S	0.117	0.765
N	0.429	0.25
I	0.452	0.222
T	0.067	0.865

BMO, bruch's membrane opening; ONH, optic nerve head; RNFL, retinal nerve fiber layer; VF, visual field; MD, mean deviation;

▲Statistical analysis is calculated with Pearson correlation analysis or Spearman correlation analysis
